# Non-Linear Relationships between Aflatoxin B_1_ Levels and the Biological Response of Monkey Kidney Vero Cells

**DOI:** 10.3390/toxins5081447

**Published:** 2013-08-14

**Authors:** Reuven Rasooly, Bradley Hernlem, Xiaohua He, Mendel Friedman

**Affiliations:** 1 Foodborne Contaminants Research Unit, Agricultural Research Service, USDA, Albany, CA 94710, USA; E-Mails: bradley.hernlem@ars.usda.gov (B.H.); xiaohua.he@ars.usda.gov (X.H.); 2 Produce Safety and Microbiology Research Unit, Agricultural Research Service, USDA, Albany, CA 94710, USA; E-Mail: mendel.friedman@ars.usda.gov

**Keywords:** aflatoxin B_1_, Vero cells, bioactivity, toxicity, non-linear dose effect, milk, meat, MTT assay, GFP assay, food safety

## Abstract

Aflatoxin-producing fungi contaminate food and feed during pre-harvest, storage and processing periods. Once consumed, aflatoxins (AFs) accumulate in tissues, causing illnesses in animals and humans. Most human exposure to AF seems to be a result of consumption of contaminated plant and animal products. The policy of blending and dilution of grain containing higher levels of aflatoxins with uncontaminated grains for use in animal feed implicitly assumes that the deleterious effects of low levels of the toxins are linearly correlated to concentration. This assumption may not be justified, since it involves extrapolation of these nontoxic levels in feed, which are not of further concern. To develop a better understanding of the significance of low dose effects, in the present study, we developed quantitative methods for the detection of biologically active aflatoxin B_1_ (AFB1) in Vero cells by two independent assays: the green fluorescent protein (GFP) assay, as a measure of protein synthesis by the cells, and the microculture tetrazolium (MTT) assay, as a measure of cell viability. The results demonstrate a non-linear dose-response relationship at the cellular level. AFB1 at low concentrations has an opposite biological effect to higher doses that inhibit protein synthesis. Additional studies showed that heat does not affect the stability of AFB1 in milk and that the Vero cell model can be used to determine the presence of bioactive AFB1 in spiked beef, lamb and turkey meat. The implication of the results for the cumulative effects of low amounts of AFB1 in numerous foods is discussed.

## 1. Introduction

The aflatoxins are a group of mycotoxins produced primarily by *Aspergillus flavus* and *Aspergillus parasiticus* that frequently contaminate animal feed and human food, causing illness and death to consumers [1]. For example, two large outbreaks of acute aflatoxicosis in humans were reported in Kenya in 2004 [2] and in India in 1974 [3], resulting in 317 recognized human cases and 125 deaths and 397 human cases and 106 deaths, respectively. 

After it enters the cell, aflatoxin B_1_ (AFB1) is metabolized in the endoplasmic reticulum to an active epoxide and to hydroxylated forms and glucuronide and sulfate conjugates. The epoxide then undergoes spontaneous hydrolysis to AFB1-8,9-dihydrodiol, which can bind to essential proteins and enzymes and can react with DNA, forming DNA adducts. These cellular and molecular events can lead to the genesis of cancer, especially of the liver [4,5,6]. 

Extensive literature exists on the multi-faceted aspects of AFB1, as indicated by 9500 and 14,500 citations in the PubMed and Scopus databases, respectively. Because different commercial food categories have been reported to contain measurable amounts of AFB1, the question arises as to whether consumption of food containing low amounts of AFB1 over a prolonged time period can lead to liver cancer. To place the present study in proper perspective, we will first mention several recent studies on the reported AFB1 content of widely consumed food, as well as on reported incidences of liver cancers in humans that may be associated with AFB1-contaminated food.

Different food categories have been reported from various studies to contain measurable levels of AFB1: corn, wheat and nut products [7], rice and mung beans [8], peanuts [9], peanuts, maize and rice [10], corn and wheat silage [11], maize, peanut meal and poultry feed [12], peanuts, pistachio, cornflakes, maize, wheat, raisins and figs [13], spices [14], milk [15], eggs, raw milk and breast milk [16], eggs, milk and meat products [17] and animal-derived food [18]. Yard *et al*. [19] mention that aflatoxins contaminate ~25% of agricultural products worldwide. These authors detected serum AFB1-lysine adducts in 78% of 600 Kenyan specimens, with a highest value of 7.87 pg/mg albumin, suggesting that aflatoxin exposure is a public health problem in Kenya that could impact human health. Almeida *et al*. [20] found that the highest level of AFB1 contamination in 230 rice products sold in Brazil were found in sub-products derived from rice bran and husk. A related study by Jager *et al*. [21] found that although there were only low levels of AFB1 in a variety of Brazilian food samples, the evaluated foods may contribute significantly to the overall human exposure. A Canadian study [22] found that 56% of 200 evaluated rice samples contained AFB1, ranging from 1.44 to 7.14 ng/g for five of the most contaminated samples. A Malaysian study [23] found an aflatoxin content of 65.4% for 55 marketed foods, ranging from 620 to 1,670 μg/kg. An Indian study of 1,646 samples of wheat grain [24] found that 40.3% of the samples contained AFB1 with a maximum level of 606 μg/kg, suggesting that the calculated daily intakes of wheat-based AFB1 were much higher than the suggested provisional maximum tolerable daily intake. A Chinese study [25] found that AFB1 was the predominant toxin in 36 corn and wheat samples from local markets, with concentrations ranging from 0.42 to 3.41 μg/kg. A survey of food sold in Japan revealed that 10 of 21 peanut butter and 22 of 44 chocolate samples contained aflatoxins, with the highest value of 2.59 μg/kg present in peanut butter. The published data show a wide-ranging AFB1 content in widely consumed food sold in different countries.

Our recent review of the literature suggests that food-compatible natural compounds and plant extract can be used to reduce the AFB1 content of food and its adverse *in vivo* effects [6]. In another study, we found that low levels of AFB1 stimulate monkey Vero kidney cells, whereas high levels kill the cells [26]. This finding raises two important questions about AFB1 toxicity: (a) what are the biological consequences of the consumption of low, possibly nontoxic, levels of AFB1 that may still be present in food after exposure to inactivation by various methods; and (b) does the continuous consumption of AFB1 elicit a cumulative, toxic effect or not? 

To help find answers to these questions, we will first mention selected recently reported studies on AFB1-induced cancers in humans.

- The incidence of hepatocellular carcinoma (HCC) is significantly elevated in the Hispanic community in Bexar County, Texas [27]. The AFB1-lysine adduct level detected in 20.6% of evaluated individual serums ranged from 1.01 to 16.57 pg/mg. Aflatoxin M1 (AFM1), a metabolite of AFB1, detected in 11.7% of the urine samples ranged from 1.89 to 935.5 pg/mg creatinine. AFM1 levels were associated with increased consumption of corn tortillas, nuts and rice.
- On the basis of a review of the literature, Matsuda *et al*. [28] suggest that mycotoxins represent risk biomarkers for nonviral hepatocellular carcinoma, a common malignant disease with poor prognosis.
- Ghasemi-Kebria *et al*. [29] found a positive relationship between the aflatoxin content of 100 wheat flour samples in areas of Iran and a high risk of esophageal cancer.
- Hamid *et al*. [30] report that about 4.5 billion of the world’s population is exposed to aflatoxin-contaminated food, especially in low-income countries, and that dietary exposure to aflatoxins is a major HCC risk factor.
- Sun *et al*. [31] reported that on the basis of the measured content of AFB1 and fumonisin B_1_ of 209 food samples in three areas of China, co-exposure to the two mycotoxins in rural China seems to contribute to the etiology of human chronic diseases in high-risk areas.
- Asim *et al*. [32] found that, in comparison with high-aflatoxin-exposure countries, such as China and Taiwan, the aflatoxin level in India, as well as the hepatocellular carcinoma rate is relatively low and that high hepatitis B virus (HBV)-DNA serum levels increased the risk of liver cancer.
- In another study, Asai *et al*. [33] reported that red chili peppers from Bolivia and Peru contaminated with aflatoxins at concentrations above maximum levels in spices proposed by the European Commission are consumed by populations that have a high incidence of gallbladder cancer.

The following selected observations indicate that animals are also susceptible to the toxic effects of aflatoxins:

- Dairy animal feed contamination by AFB1 near the European Union tolerance of 5 μg/kg results in a concentration of AFM1 in milk higher than the European Commission maximum tolerance level, suggesting that AFM1 in milk may originate from dairy cows [34].
- On the basis of a study that showed that AFB1 in cows is carried over to AFM1 in milk, the authors suggest that the maximum AFB1 level in feed should not exceed 1.4 μg/kg, a value 3.6-times lower than the maximum residue level currently applied in Israel [35].
- A new stable isotope dilution assay was used to detect the AFMI content of condensed milk, milk-based infant formula and table cream imported into the United States [36].
- An outbreak of aflatoxin poisoning in 65 dogs was associated with two corn meals containing 1640 ppb and 1770 ppb of AFB1, respectively [37].
- Duck mortality increased with increasing concentrations of AFB1 in the diets [38].
- AFB1 produced dose-related DNA damage in fetal livers of both chicken and turkey ova, with turkey embryos showing slightly more susceptibility to AFB1 damage than chicken embryos [39].
- Hepatic cytochrome P450 1A5 is the dominant enzyme for AFB1 bioactivation and metabolism of environmentally-relevant AFB1 concentrations in turkey liver [5].
- *Bacillus subtilis* bacteria ameliorated damage of liver and kidney tissue in laying hens exposed to AFB1 [40].
- Consumption of AFB1-contaminated feed by cattle reduces growth rate, decreases the efficiency of converting feed into animal protein and decreases milk production [41,42].

One important characteristic of aflatoxins is their capacity for bioaccumulation and bioconcentration; therefore, even extremely low levels of aflatoxins in feed can become significant over the lifetime of an animal. The information in the cited references seems to indicate that the Food and Drug Administration (FDA) temporarily allowed the blending of grain containing higher levels of AFB1 with animal feed containing lower levels as a relief measure for farmers affected by highly contaminated grain in 2012 [43]. According to the Food and Agricultural Organization (FAO) of the United Nations, feed containing significant amounts of aflatoxin should not be fed to food-producing animals [44].

Because we previously reported [26] that, at low levels, AFB1 induced cellular activity of the Vero cells, whereas at higher levels, the toxin killed the cells, the main objective of the present study was to further define the apparent non-linear nature of the dose response at the cellular level induced by several AFB1 concentrations and the biological activity in the Vero cells. As part of this effort, we also attempted to find out whether Vero cells can be used as a model for determining the bioactive AFB1 content of spiked milk, beef and poultry products.

## 2. Results

### 2.1. Low Doses of AFB1 Stimulate Cell Growth and Activity

Although it has been shown that consumption of high levels of AFB1 has a potent toxic and carcinogenic effect, lower concentrations of AFBs are allowed in animal feed, presumably because low concentrations of the toxin are considered safe with limited biological activities. To study the effect of low concentrations of AFB1 on the biological cell activities of Vero cells, we quantitatively measured the biological activities of the cells exposed to several concentrations of the toxin. 

The viability of the cells was measured using the tetrazolium salt (MTT) assay, a widely used colorimetric method for measuring the cellular reduced pyridine nucleotide cofactor, NADH, which is responsible for most tetrazolium salt (MTT) reduction [45].

**Figure 1:**
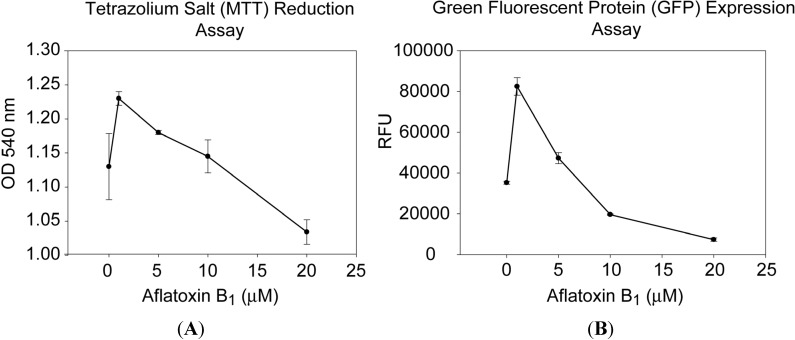
Increase in tetrazolium salt (MTT) reduction (**A**) and green fluorescent protein (GFP) expression (**B**) in Vero cells in the presence of low concentrations of aflatoxin B_1 _(AFB1). Vero cells (**A**) and Vero cells transduced with Ad-CMV-GFP (**B**) were treated with increasing concentration of AFB1. The MTT reduction was colorimetrically measured, and GFP expression was quantified fluorometrically. Error bars represent standard errors (*n* = 3).

As shown in Figure 1a, the biological activities of the cells measured by MTT assay peaked after exposure to a low concentration of 1 μM of AFB1. In contrast, the biological activity decreased at higher concentrations of 5, 10 and 20 μM of AFB1. We found similar results when we generated and used adenoviral vectors that encode and express the green fluorescent protein (GFP) gene (Ad-GFP) and used the GFP fluorescent intensity in transduced cells for quantitative measurement of the biologically active aflatoxin B_1_. As shown in Figure 1b, at 1 μM of AFB1, there was increased GFP fluorescent intensity compared with the control untreated cells. By contrast, at higher concentration of 5, 10 and 20 μM of AFB1, there was a significant negative relationship between GFP fluorescent intensity and AFB1 concentration. The level of GFP fluorescent intensity decreased in a dose-dependent manner. These paradoxical results suggest that there is a non-linear dose response relationship between AFB1 concentrations and biological activity. Low levels of AFB1 resulted in the highest cellular activity, but higher levels had an opposite effect. Therefore, we cannot extrapolate and predict the effect of low levels of toxin by using higher concentrations, at least for the Vero cells. 

### 2.2. Heat Treatment Did Not Affect AFB1 Stability in Milk

**Figure 2:**
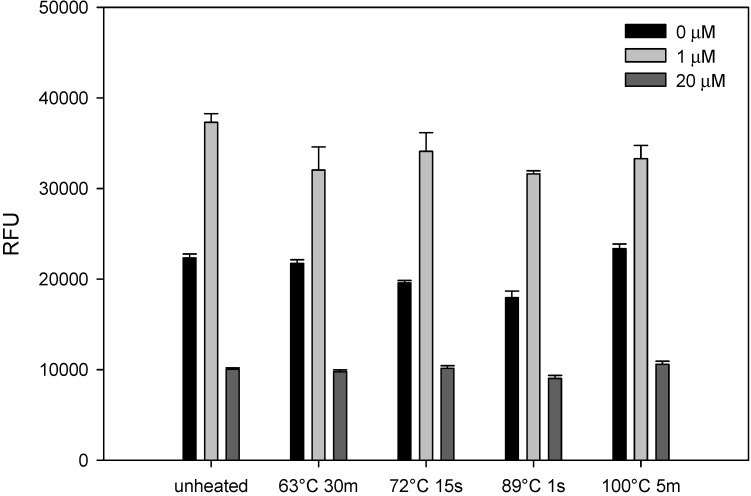
Detection of AFB1 in milk. Nonfat dry milk was spiked with AFB1 at 1 and 20 μM. Five microliters of unheated or thermally-treated spiked milk with 95 μL of media were pre-incubated for 48 h in Vero cells. The cells were then transduced with Ad-CMV-GFP for 48 h. GFP expression was quantified fluorometrically, with the plot showing relative fluorescence units (RFU). Error bars represent standard errors (*n* = 3).

Dairy cattle that ingested contaminated feed with nonfatal levels of AFB1 produce milk contaminated with the carcinogenic AFB1 and its hydroxylated metabolite, aflatoxin M1 [35,41]. We therefore tested whether thermal treatment would inactivate the toxin in milk and evaluated the ability of the assay to detect active AFB1 in milk. Milk was spiked with AFB1 at concentrations of 1 μM and 20 μM, pasteurized at 63 °C for 30 min, 72 °C for 15 s, 89 °C for 1 s and, then, subjected to thermal treatment at 100 °C for 5 min. We used transduced Vero cells with Ad-GPF for the bioassay to detect biologically active AFB1 in milk (Figure 2). This figure shows that there was no linear dose-response relationship between AFB1 concentrations and GFP fluorescent intensity. When milk was spiked with low concentration (1 μM) of AFB1 and added to the cells, the transduced cells emitted higher fluorescence intensity. The spiked milk with 20 μM AFB1 statistically reduced GFP fluorescent intensity compared with the control, unspiked milk (*p* < 0.05). These results show that AFB1 is heat-stable, is not inactivated by pasteurization and that the toxin retains its activity in milk, even after thermal treatment at 100 °C for 5 min. 

### 2.3. Detection of AFB1 in Beef, Lamb and Turkey Meat

**Figure 3:**
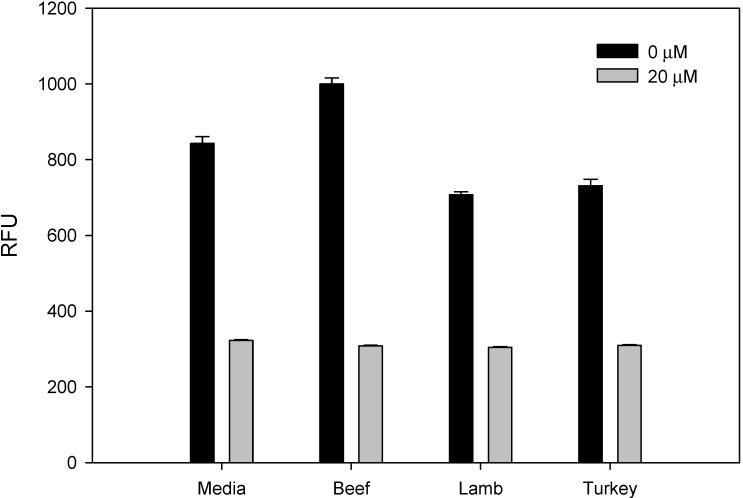
Detection of AFB1 in meat. Beef, lamb and turkey were spiked with AFB1 at a concentration of 20 μM. Five microliters of the spiked meat with 95 μL of media were pre-incubated for 48 h in Vero cells. The cells were then transduced with Ad-CMV-GFP for 48 h, as described. GFP expression was quantified fluorometrically, with the plot showing relative fluorescence units (RFU). Error bars represent standard errors (*n* = 3).

As mentioned above, consumption of low levels of AFB1 increases the risk factors for liver cancer in humans. To evaluate the potential of the assay to detect AFB1 in beef, lamb and turkey, these meats were spiked with 20 μM AFB1. The spiked meats were added to Vero cells, and after incubation for 48 h, the cells transduced with Ad-GFP and GFP expression were measured fluorometrically after another 48 h. As shown in Figure 3, in spiked meat, AFB1 statistically reduced GFP fluorescent intensity expression compared with unspiked meat (*p* < 0.05). These results suggest that this diagnostic method has the potential to be used for detection of bioactive AFB1 in a variety of meat products. A practical, inexpensive assay could be developed after more extensive dose-response and multiple sampling studies.

## 3. Discussion

High performance liquid chromatography (HPLC) and enzyme linked immunoassays (ELISA) are the main analytical methods that can be used to determine the AFB1 content of food. Neither assay can, however, distinguish between the biologically active forms of the toxin, which pose a threat to life, and the inactive form. In this study, we developed quantitative methods for the detection of biologically active AFB1 in food. This assay allows the visualization of the activity of AFB1 on living cells without added substrates or using cell fixation methods. AFB1 at concentrations higher than 1 µM inhibits GFP protein synthesis in a dose-dependent manner. Bioactive AFB1 reduces the GFP fluorescence intensity emission in a dose-dependent manner. 

This result was confirmed by a second assay that measures the mitochondrial dehydrogenase activity. This reduction assay uses MTT to measure cellular metabolic activity via NAD(P)H-dependent cellular oxidoreductase enzymes. In this study we demonstrated by both assays that there is a non-linear dose-response relationship of AFB1 at the cellular level. 

The suggested use of Vero cells to determine the presence of bioactive AFB1 in feed and food is supported by the reported observations that (a) the combinations of AFB1 and ochratoxin A induce an additive apoptosis in the Vero cells [46] and (b) exposure of the Vero cells to UV radiation transforms AFB1 to a more toxic product [47]. 

Our results show that AFB1 at a concentration of 1 µM has an opposite effect to that of higher levels, which inhibit metabolic activity and protein synthesis. We speculate that low concentrations of AFB1 stimulate an adaptive response in the cell that increases cellular activity and alters cell physiology to increase levels of protective molecules and enhances the capability of cellular systems involved in adaptation and cell survival. Exposure to higher levels of AFB1 damages the cell beyond its limit of recovery, and it cannot overcome the damaging effect, resulting in cell death. We, therefore, cannot extrapolate and predict the effect of low levels of AFB1 from the observed effects of higher concentrations.

The apparent decision of the FDA to permit temporary blending of AFB1-contaminated grain with uncontaminated grain for use as animal feed [43] implicitly assumes that the deleterious effects of AFB1 are positively correlated to the final concentration and that levels of AFB1 in feed that are not toxic to the animal are of no further concern. This assumption may be invalid, even for the toxicity effect alone, because of the capacity for AFB1 bioaccumulation and bioconcentration of AFB1 *in vivo* [48] and in cells [49]. 

The possible bioaccumulation of low concentrations of AFB1 in humans, here defined as a dose lower than that causing aflatoxicosis, is supported by previously mentioned acute outbreaks that occur regularly in some countries, especially China, India, Kenya and other African countries, where many people consume lower than acute doses over a lifetime. Dietary AFB1 seems to be a major risk factor of hepatocellular carcinoma in these countries. Low levels of AFB1 in feed and food can become significant over the lifetime of an animal and contaminate cereal, meat and other food products. Previous efforts to relate dose-response in carcinogenesis of AFB1 include the following observations:

- On the basis of a review of more than 500 National Toxicology Program Technical Reports, Waddell [50] concluded that the mere presence of DNA adducts of AFB1 does not necessarily lead to tumor formation, that all of the carcinogenicity studies show a linear response when the dose is on a logarithmic scale, that the concept of hormesis, defined as a dose-response effect characterized by low-dose stimulation and high-dose inhibition [51], probably applies to carcinogenesis and that a cumulative dose may be a better indicator than a daily dose.
- On the basis of simulation studies, Lutz *et al*. [52] concluded that non-linear dose-response curves are often observed in tests of carcinogenicity in rodents and that linear extrapolation of a human risk could be justified, even if animal bioassays show non-linearity.
- During a long-term study, Jossé *et al*. [49] found that AFB1 induced a dose-dependent cumulative cytotoxicity in human HepRG hepatocytes, suggesting that these cells represent an *in vitro* liver cell model for measuring acute and chronic toxicity and genotoxicity of AFB1 in human livers.
- Williams *et al*. [53,54] observed nonlinearities and thresholds in rat liver carcinogenesis caused by 2-acetylamianaofluorene and diethyl nitrosamine. The authors interpret the results to reflect thresholds for the initiation of liver cancer by these carcinogens and an exaggerated response at high exposures, due to toxicity and compensatory hepatocyte proliferation. The possibility, therefore, exists of defining safe low exposure levels (SEL) to carcinogens.
- Using the MTT assay, Ruiz *et al*. [55] investigated the cytotoxicity in Vero cells induced by individual and combinations of three *Fusarium* toxins (beauvericin, deoxynivalenol and T-2 toxin). All combinations exhibited antagonistic effects. The highest antagonistic results were obtained with the binary mixture of deoxynivalenol and the T-2 toxin, suggesting the need to evaluate the potential toxicities of combinations of mycotoxins that may be present in some food. By contrast, Bouaziz *et al*. [56] found that mixtures of the fusarial toxins, zearalenone and T-2, induced higher cytotoxicity in Vero cells, suggesting that each combination of mycotoxins needs to be evaluated for toxicity.
- Using membrane-engineered Vero cells, Larou *et al*. [57] developed a rapid 3-min biosensor assay for AFM1, suggesting the need to determine the applicability of the assay to mycotoxin-containing food.

## 4. Materials and Methods

### 4.1. Materials

Aflatoxin B_1_ (AFB1) and 3-(4,5-dimethylthiazol-2-yl)-2,5-diphenyltetrazolium bromide (MTT) were purchased from Sigma-Aldrich (St. Louis, MO, USA). Human Embryonic Kidney 293 cells (HEK293) (ATCC CRL-1573) and Vero African Green Monkey adult kidney cells (ATCC CCL-81) were obtained from American Type Culture Collection (Manassas, VA, USA).

### 4.2. Effect of Heat on AFB1 Bioactivity in Milk

To determine the effect of heat on AFB1 activity, samples of Dulbecco’s Modified Eagle Medium (DMEM) (500 µL) spiked with increasing concentrations of AFB1 in tightly screw-capped Eppendorf tubes (1.5 mL) were inserted in an Eppendorf Dry Heating Block (Government Scientific Source Inc., Reston, VA, USA) set at 63 °C, 72 °C, 89 °C and 100 °C. The cooled samples were then incubated with Vero cells. 

### 4.3. Cell Culture

Vero cell and Human Embryonic Kidney 293 cells (HEK293) were maintained in DMEM medium containing 10% fetal bovine serum (FBS) and 100 units/mL of both penicillin and streptomycin. Cells were trypsinized when ready to harvest.

### 4.4. Plaque Assays for the Purification and Titration of the Adenovirus

Plaque assays depend on the ability of the adenovirus to propagate in HEK293 cells. Six 35 mm tissue culture plates were seeded with HEK293 cells. The cells were incubated at 37 °C in a 5% CO_2_ incubator until they were 90% confluent. Serial dilutions were made in DMEM medium supplemented with 2% FBS. The diluted virus was added to the cells. After 2 h, the medium was removed and replaced with 1× Modified Eagle Medium and 1% SeaPlaque^®^ agarose (FMC Corporation, Rockland, ME, USA). The agar overlay was added to keep the virus localized after the cells had lysed. After 5 days, plaques were visible and were counted for titer determination after 7 days.

### 4.5. Quantifying Recombinant Adenovirus Expressing Vectors that Encode the GFP Gene

Vero cells were plated on black 96-well plates (Greiner 655090 obtained from Sigma) at 1 × 10^4^ cells in 100 µL of medium per well. Cells were incubated overnight to allow time for cells to attach to the plate. Spiked samples (either 15 µL of sample in 85 µL of media or 100 µL of media spiked with toxins) were then added to each well and incubated for 48 h at 37 °C in a 5% CO2 incubator. The cells were then transduced with Ad-GFP at a Multiplicity of Infection (MOI) of 100 for 48 h. The media was removed, and cells were washed three times with pH 7.4 phosphate buffered saline (PBS). 

Quantification of fluorescence emission by the cells expressing GFP was measured using a 528/20 nm emission filter and 485/20 nm excitation filter in a Synergy HT Multi-Detection Microplate Reader (BioTek, Winooki, VT, USA). 

### 4.6. MTT Assay for the Metabolic Activity and Viability of Cells

Vero cells were plated on black 96-well plates (Greiner 655090) at 1 × 104 cells in 100 μL of medium per well. The cells were incubated overnight to allow time for the cells to attach to the plate. Spiked and unspiked control 100 μL medium were added to separate well sand incubated for 9 h at 37 °C in a 5% CO_2_ incubator. Methylthiazolyldiphenyl-tetrazolium bromide (MTT) was diluted in PBS to 2 mg/mL, and 25 μL was added to each well. Plates were incubated at 37 °C for 4 h, and the medium was removed. Viable cells reduce the yellow, water-soluble MTT reagent to a purple formazan salt, which is water soluble and can be colorimetrically detected. To each well, 100 μL of dimethyl sulfoxide (DMSO) was added, and plates were read at 540 nm. 

### 4.7. Statistical Analysis

Statistical analysis was performed with SigmaStat 3.5 for Windows (Systat Software, San Jose, CA, USA). Multiple comparisons among transduced-treated cells were made. One-way analysis of variance (ANOVA) was used to compare transduced-treated cells (containing increasing concentrations of toxin or milk) to control and transduced-untreated cells. The experiments were repeated at least three times, and results with *p* < 0.05 were considered statistically significant.

## 5. Conclusions

In summary, our results show that even at a low concentration, AFB1 enhances cellular activity. We previously observed a similar biphasic concentration response during the inhibition of *Salmonella* bacteria by the olive compound, 4-hydroxytyrosol [58,59]. We do not know the biochemical basis and possible adverse effects of these low-dose activities by a fungal toxin and virulent bacteria. A journal referee suggested that the possibilities for the observed putative adaptation mechanism of Vero cells at low levels of AFB1 could be the result of changes in reactive oxygen species (ROS) in the cells, a well-established part of adaptive responses, as well as to the induction of heat-shock HsP70 proteins [56]. These and other mechanistic aspects of the low-dose effects of mycotoxins merit further study.

One possible beneficial effect of the enhanced activity induced by low doses of AFB1, ricin and milk is the stimulation of the biosynthesis of recombinant proteins in the Vero cells [26]. The observed non-linear dose response results may have implications for the use of blended grain containing higher levels of AFB1 for use as animal feed and for human consumption of food with low AFB1 content, because it is likely that the residual low aflatoxin levels may have unknown biological effects. The consequences of consuming feed or food with low AFB1 content or agricultural products produced from animals raised on such feed, the cumulative aspects of AFB1 consumption by humans and the use of Vero cells to determine the presence of bioactive AFB1 and AFM1 in food merit further study.
